# Functional Coronary Revascularization: A Valuable Underutilized Diagnostic Approach

**DOI:** 10.31083/j.rcm2512430

**Published:** 2024-12-05

**Authors:** Lloyd W Klein

**Affiliations:** ^1^Cardiology Division, University of California, San Francisco, CA 94143, USA

Physiological testing of coronary artery stenoses enhances diagnostic accuracy and improves clinical outcomes by providing a more accurate assessment of which stenoses are severe, and thus which patients are likely to benefit from revascularization. By avoiding unnecessary interventions and focusing treatment on those lesions that are truly hemodynamically significant, better patient outcomes and more efficient use of healthcare resources results. The standard evaluations of physiological significance are fractional flow reserve (FFR) and instantaneous wave-free ratio (iFR). Deferring revascularization based on normal FFR/iFR results is cost-effective and leads to favorable long-term clinical outcomes [1, 2, 3]. Functional assessment, when used in conjunction with anatomic severity estimates, has been linked to better long-term outcomes in angiographically intermediate lesions [4]. These physiological evaluations can play a vital role in influencing the decision-making process, aiding in the selection of coronary revascularization procedures, and optimizing procedural outcomes.

Although physiologic lesion assessment has been increasingly utilized in clinical practice, its overall utilization remains low [5]. Landmark trials have highlighted the safety, cost-effectiveness, and reduction in adverse cardiac events associated with an FFR-guided revascularization strategy compared to the angiography-guided approach. About 70% of patients referred for percutaneous coronary intervention (PCI) have multivessel coronary artery disease (CAD) (multivessel disease, (MVD)) [4], a group in which non-invasive testing is often unreliable in differentiating severe from non-obstructive disease in a particular vessel. FFR/iFR is especially valuable when there is a discrepancy between lesion severity and non-invasive findings.

Incorporating the results of these tests into the evaluation of stenosis severity may alter the angiographic estimate of the number of vessels diseased. Increased diagnostic accuracy might lead to a modification in the decision regarding revascularization and may alter the type and extent of the revascularization procedure to be performed. The SYNTAX score is an angiographic scoring system to grade the complexity of coronary artery stenoses. The morphologies classified and the point system used were derived from a combination of the American Heart Association (AHA) classification for coronary segments, a modified Leaman score, the American College of Cardiology (ACC)/AHA lesion classification system, a combination of the Duke and Institut Cardiovasculaire Paris Sud (ICPS) bifurcation classification system, a chronic total occlusion classification system, and expert opinion. The SYNTAX II score blends anatomic and clinical prognostic variables. These scores produce relatively accurate mortality estimates to guide the selection of PCI vs. coronary artery bypass grafting (CABG) [1, 2, 3, 4, 5, 6, 7].

When SYNTAX scores are modified by physiologic evaluation, a Functional SYNTAX score (FSS) is generated, which has the potential to reclassify the number of vessels diseased, and the lesions of significance, identified through angiography. This reclassification might then impact the choice of medical therapy alone, PCI or CABG. The effectiveness of this approach was tested by comparing the outcomes of SYNTAX II and SYNTAX I. In SYNTAX II, iFR was performed in 74% of the lesions, resulting in the deferral of treatment in 31% of the lesions that were examined. After one year, the outcomes of PCI using this treatment strategy were found to be similar to those of the CABG cohort in SYNTAX I. However, the PCI cohort had lower major adverse cardiac event (MACE) scores compared to the PCI cohort in SYNTAX I, with a hazard ratio of 0.58 (95% CI 0.39–0.15). At 1 year, PCI with this treatment strategy had similar outcomes to the CABG cohort in SYNTAX I. The PCI cohort, however, had lower MACE scores compared to the SYNTAX I PCI cohort (HR 0.58, 95% CI 0.39–0.85). This approach may be especially relevant when appraising lesion severity when no stress tests are available or when the angiographic interpretation and stress test findings are discordant [4, 5, 6, 7, 8, 9, 10].

For these reasons, the appropriate utilization of these examinations remains uncertain: should they be employed in specific instances based on clinical judgment or in all patients undergoing coronary angiography? The RIPCORD trial [11], a study involving 1100 patients, found no significant disparity in in-hospital expenses and quality of life at the one-year mark when comparing angiography alone to angiography combined with FFR testing in all cases. The routine use of FFR did not result in cost reduction, enhancement of quality of life, or a decrease in major adverse cardiac events or rates of revascularization in comparison to angiography alone. However, it was linked to elevated rates of complications, contrast utilization, and procedural duration. In real-world contemporary practice [12], implementing invasive physiological testing in MVD patients strongly influences the preferred course of treatment, leading to a notable rise in the selection of PCI. Patients appear to be chosen for functional testing based on whether the results might prompt a greater inclination toward PCI. Since ascertaining objectively which stenoses can produce ischemia may alter the preferred treatment strategy, the results of these modalities result in a significant increase in the utilization of PCI.

Non-invasive testing is often unreliable in MVD in identifying which stenoses are ischemia-producing, and which are not, a limitation that functional testing resolves. These determinations are crucial to selecting the best treatment strategy. The FAME-2 trial [4] demonstrated the superiority of FFR-guided PCI plus medical therapy over medical management alone in MVD. Additionally, FAME-3 compared the outcomes of FFR-guided PCI vs. CABG in patients with three vessel disease (3VD) [13]. FFR-guided PCI was not found to be inferior to CABG with regards to incidence of death, MI, stroke, or repeat vascularization at 1 year. This difference was largely driven by a greater need for urgent revascularization in patients managed with medical therapy alone. These findings raise questions about when to employ physiologic parameters in selecting patients for CABG, particularly when there is anticipation that non-significance in one or more vessels would divert a patient from open-heart surgery and instead receive coronary stenting.

In this issue of Reviews in Cardiovascular Medicine, Zhang and colleagues [14] analyzed the results of quantitative flow ratio (QFR), a non-invasive physiologic assessment of fractional flow reserve based on contrast passage in 160 MVD patients. The study presents a comparative analysis of the Functional SYNTAX Score II (FSSII) and SYNTAX Score II (SSII) as predictors for MACE in MVD. They found that a large proportion of patients had less extensive disease, and hence lower risk, when functional testing was added to routine angiographic analysis. For SS, 20% of cases (32/160) were re-classified downward in risk, and for SSII, about 4%. As shown in Fig. 2 & Table 4, FSS compared with SS, and FSII compared with SSII, improved the prediction of MACE. The survival-free ratio stratified by FSSII decreased from 38.46% to 27.27% in the high-risk group and increased from 84.09% to 86.05% in the low-risk group. These data emphasize the importance of FSSII as a reliable tool for risk assessment and treatment decision-making in MVD, confirming the value of functional testing.

The findings also suggest that physiological testing should probably be utilized in all cases rather than selectively because there is no reliable way to predict which lesions are systematically overestimated by angiography. Operator-determined patient selection introduces a cognitive bias in favor of PCI. Cardiologists should never accept diagnostic error as part of clinical practice. Complacency with “how things have always been done” has no place when patients are involved. However, appropriate concerns include that invasive assessments are associated with higher costs, longer time requirements, and increased utilization of resources. The upfront cost of the monitor and the disposable flow wires used in FFR and iFR can be substantial. Additionally, reimbursement policies for these procedures can vary, which may deter their routine use in certain healthcare settings. Adding physiological assessment to coronary angiography can increase the duration of the procedure. This can be a deterrent in busy clinical settings where time efficiency is crucial. For routine adoption, these barriers would need to be addressed through improved training, cost management, better resource allocation, and evolving clinical practice guidelines [3].

It may seem counterintuitive to think that adding another procedure could decrease the overall cost of care, but the answer lies in reducing the use of CABG, an expensive revascularization procedure. Our surgical colleagues for this reason tend not to rely on these tests and have not identified cut-off values to improve case selection. Prospective randomized trials need to be conducted to evaluate clinical outcomes following a similar approach for CABG as demonstrated in this small-scale study [14]. Since angiography frequently overestimates stenosis severity, fewer patients may require CABG, a more costly revascularization procedure. This deduction suggests that these tests are not an additional expense for providing optimal health care, but rather “costly” to the hospital, which would experience a decrease in profit. When appraising costs in healthcare, one must always ask: the cost to whom?
